# Preferential Root Tropisms in 2D Wet Granular Media with Structural Inhomogeneities

**DOI:** 10.1038/s41598-019-50653-8

**Published:** 2019-10-02

**Authors:** Cesare M. Cejas, Lawrence A. Hough, Raphaël Beaufret, Jean-Christophe Castaing, Christian Frétigny, Rémi Dreyfus

**Affiliations:** 1 Complex Assemblies of Soft Matter, CNRS-Solvay-UPenn UMI 3254, Bristol, PA 19007-3624 USA; 2 Sciences et Ingénierie de la Matière Molle (SIMM) CNRS UMR 7615 ESPCI, 10 rue Vauquelin, Paris, 75005 France; 3 Present Address: Microfluidics, MEMS, Nanostructures Laboratory, CNRS Chimie Biologie Innovation (CBI) UMR 8231, Institut Pierre Gilles de Gennes (IPGG), ESPCI Paris, PSL Research University, 6 rue Jean Calvin, Paris, 75005 France

**Keywords:** Drought, Hydrology

## Abstract

We investigate certain aspects of the physical mechanisms of root growth in a granular medium and how these roots adapt to changes in water distribution induced by the presence of structural inhomogeneities in the form of solid intrusions. Physical intrusions such as a square rod added into the 2D granular medium maintain robust capillary action, pumping water from the more saturated areas at the bottom of the cell towards the less saturated areas near the top of the cell while the rest of the medium is slowly devoid of water via evaporation. The intrusion induces “preferential tropism” of roots by first generating a humidity gradient that attracts the root to grow towards it. Then it guides the roots and permits them to grow deeper into more saturated regions in the soil. This further allows more efficient access to available water in the deeper sections of the medium thereby resulting to increased plant lifetime.

## Introduction

Root-water interactions remain an important topic in understanding transport to maintain robust water absorption in the face of today’s depleting resources. Such interactions are complex owing to the sensitivity of root systems and the presence of a wide variety of factors that affect not just large-scale (ecosystem level)^1,2^ but also small-scale dynamics (sub-organismal or cellular)^2–4^. Fundamentally, research involving root systems are narrowed down in terms of physical (e.g. soil particle type or size), chemical (e.g. effect of additives), and/or biological (e.g. hormone responses) variables. Root elongation is sensitive to external factors^5^. They evidently respond to gravity (gravitropism)^6^, where fluorescence studies focus on molecular interactions that govern macroscopic root reflexes such as bending. While light (phototropism) may not be considered a major tropism for root growth, studies^7^ have nevertheless provided evidence on oriented growth of certain types of roots based on the position of the light source. In addition, roots are also sensitive to their environment^5^. For instance, the granular nature of the soil can complicate the growth process. For this reason, studies have investigated the correlation between global root architecture and its interactions with the soil particles^1,8,9^ and water uptake^10–12^. Literature on root mechanics and root growth depend on properties of soil structure^13^ such as soil compaction^1,8^ and soil inhomogeneity^14,15^. Root mechanics requires understanding of forces that the root exerts on the granular material^16^. For this purpose, the choice of granular substrate is often photoelastic grains, where root contact with the grain triggers birefringence^17,18^, root growth in different media have also been reported such as agar^19^, sand^20^, deformable gels^16^, photoelastic grains^17^, or glass beads^21^. Roots have shown to exhibit growth in large holes (trematropism) or macropores^22,23^ in the granular medium, which are areas of low resistance. In addition to mechanical impedance, water stress also helps drive root elongation in granular medium^4^. Roots experience stress due to the minimal amount of water left in the medium^4,22^, which can decrease cell division rates^24^ and limit growth^4,22^. Studies^25–27^ have also shown that roots can sense a gradient of moisture in its environment and generally grow towards the region of higher moisture or saturation content. Thus, roots respond to water saturation (hydrotropism) as shown by growth experiments with respect to water uptake or absorption^10,11^ or evaporation and transpiration^12^. Studies have therefore shown that water stress also helps drive root elongation in granular medium^4^.

Interest in water retention has inspired research investigations on water absoprtion and root growth^11,14^, with the goal of improving water accessibility and availability for crop productivity^2^. To improve water retention, the most common method used is additives in the form of hydrogels. For this reason, some studies have focused on the dynamics of hydrogel swelling to trap water for long periods of time^28,29^. The roots are often located in an area of the soil called the vadose zone^30^, which is a partially saturated region that contains a mixture of air, hydraulic networks, and disconnected films^31–35^.

What if we can mechanically induce capillary action, such that water from the deeper and more saturated areas is consistently pumped upwards towards the root zone? This change in water distribution can be generated by modifying the structure of the granular medium by introducing inhomogeneities^14^, whose purpose is to redistribute water from the bottom (an area of higher water saturation) to the top (an area of lower saturation). Capillary action to move water from the more saturated bottom layer to the less saturated top layer is similar to hydraulic lift phenomenon^36–39^, providing interesting insights into the relationship between the root and the surrounding granular medium.

In this study, we investigate experimentally the root growth behavior in response to a change in water distribution induced by the presence of structural inhomogeneities in the granular medium. Our choice of inhomogeneity is a solid intrusion, which is inserted in the 2D granular medium. This solid intrusion extends throughout the depth of the growth cell, linking the upper portion of the medium near the surface to the fully wet zone at the bottom. We show in these experiments that root elongation can be influenced by the structural inhomogeneities inside the granular medium (“preferential tropism”), thereby inducing a change in water distribution that can significantly control direction and movement of root growth and the subsequent lifetime of a plant. The effect of the intrusion is two-fold. First, it pumps water from the bottom towards the top creating a gradient that elicits a reponse from the root to grow towards it (hydrotropism). Then, the intrusion further guides the roots towards the deeper parts of the soil.

## Results and Discussion

### Root morphology characterization in 2D cells with glass beads as growth substrates

Technical details on the growth cell are described in the Materials and Methods section. For our growth substrate, we use transparent glass beads, which permit simultaneous observation of water content over time. We use lentils (*L. culinaris*) because lentils grow at relatively rapid rates and they develop relatively simpler root systems, i.e. they possess a distinct large primary root and a few observable secondary roots. In this model set-up different from real soil, it is imperative to characterize root growth in this chosen substrate to first understand how these roots adapt to their model environment and to use this information as a reference when comparing root growth in structurally modified media.

**Figure 1:**
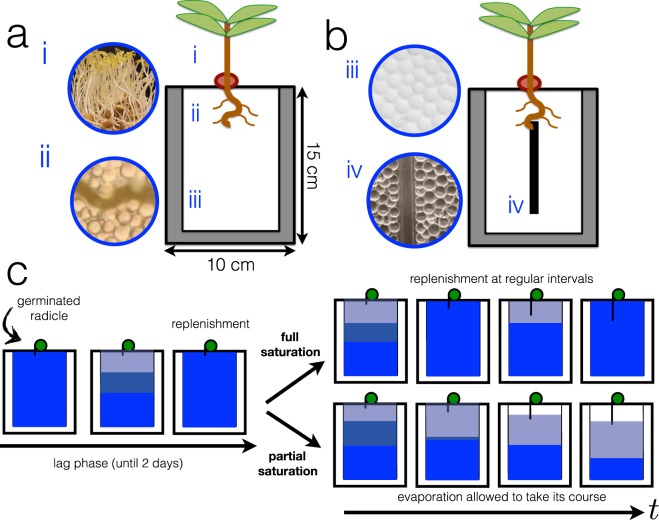
(**a**) Illustration of the 2D growth cell or rhizotron made of parallel plates with a gap of ≈1 mm. (**b**) 2D growth cell with intrusion. Inset images show the different parts of the set-up. (i) lentil shoots, (ii) lentil roots within pore spaces between glass beads, (iii) glass beads (as model soil) fully saturated with water, (iv) image of the inhomogeneity or intrusion (square rod) inserted inside the 2D granular medium. (**c**)Experimental scheme where all roots are regularly being replenished when water is lost via evaporation during the lag phase. After the lag phase, the roots may be subject to full saturation (regular replenishment) or partial saturation (no replenishment and evaporation allowed to take its course).

Root growth characterization experiments are performed under the same conditions but repeated at least three times under controlled ambient atmospheric conditions (*T* = 23° ± 2, *R*_*H*_ = 45.0 ± 5.0%), showing reproducibility of the root system in this particular 2D set-up. Once a radicle germinates from the seed, it is transplanted on top of the 2D growth cell. It is expected that after germination, root growth is normally slow because the roots are still developing the necessary biological functions dedicated for root functions. This initial slow growth phase is termed as lag phase, where radicles are most vulnerable^40^. At the start of all experiments, the 2D cells are filled with water up to the brim and thus overall water saturation, Φ, in the medium is Φ ∼ 1. During the lag phase (duration: 2 days maximum), water (mixed with nutrient solution), is constantly replenished into the 2D cell at regular intervals such that the young radicle does not experience sudden water stress via evaporation. As water evaporates, the granular system is slowly being depleted with water such that 0 < Φ < 1. This leads to the development of a partially saturated zone (PSZ), a mixture of air and water^12,31–35,41^, appearing inside the granular medium. We then characterize the root morphologies in partial saturation conditions and compare it to root growth in full saturation conditions. The scheme in Fig. 1c illustrates the difference between both conditions. In conditions of full saturation, water is constantly being replenished at regular intervals even after the initial lag phase such that the root, at any point during the experiment, does not experience severe water stress. In conditions of partial saturation, after the lag phase, the water in the medium is allowed to freely evaporate without additional replenishment.

**Figure 2:**
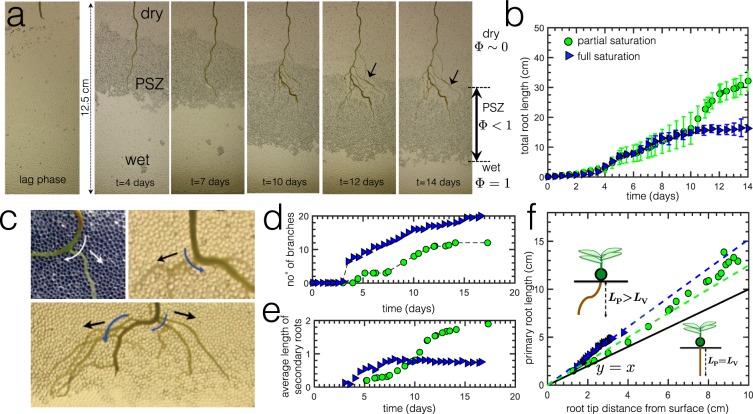
(**a**) Typical images of root growth under partial saturation conditions, where evaporation is allowed to take place after the initial lag phase. We observe three distinct zones (dry, partially saturated, wet). (**b**) Total root length as function of time for both partial saturation and full saturation conditions after the initial lag phase. Error bars reflect standard deviation. (**c**) Images showing emergence of lateral roots from convex sides (outer curves). Curved arrows signify outer curvature while straight arrows indicate growth direction. (**d**) Number of branching points as a function of time. (**e**) Average length of secondary roots normalized by the number of branching points. (**f**) Primary root length as function of root tip distance from suface. The solid line represents *y* = *x*, which corresponds to the case when the root grows in a straight vertical manner, perpendicular to the surface. () partial saturation; () full saturation.

A typical example, Fig. 2a, shows the temporal evolution of root growth as well as the PSZ evolution during evaporation in the presence of lentil root systems (partial saturation conditions). During evaporation, the PSZ initially develops around the root zone and gradually grows further inside the medium, distancing itself away from the root zone. A dry zone eventually appears just below the cell surface. The bottom of the cell remains fully wet or fully saturated. Over time, the dry zone eventually catches up and a fraction of the entire root system is exposed to this dry zone. Generally root growth rates are typically slower than water fluxes such as evaporation^10,12^ or even infiltration/drainage, and as a result, the roots are constantly under “stress” (greater quantity of water is evaporated than the quantity being absorbed)^12^, and thus the root is then quickly engulfed by the developing dry region. At this point, the root eventually dies without being able to take advantage of the considerable quantity of water left unused at the bottom of the cell, far from the root zone.

We compare the root growth in both full and partial saturation conditions, where we measure the total root length (primary + secondary). Results in Fig. 2b reveal that overall root length grows two times more in partially saturated systems, where a mixture of air and water exists. When roots experience stress, they can develop new structures^13^. The graph of total root length follows a sigmoidal pattern^40^. In Fig. 2c, we show some examples of lateral roots emerging from the convex sides of the root. Literature points out that this is related to the auxin plant hormone distribution^42^ because high concentration of auxins at the curvature of roots triggers lateral organ formation of branching points for the secondary lateral roots^6^. We also measure the average length of the secondary roots, normalized by the number of branching points emerging from the primary root. We observe that, for this root sample, tertiary roots (roots that emerge from secondary branches) do not form within the experimental observation window. Thus, the total root length mainly includes the primary and the secondary lateral roots. The graph in Fig. 2d shows that more branches grow in fully saturated conditions but Fig. 2e shows that despite the greater branching points, the resulting secondary roots under full saturation conditions ultimately have smaller lengths than in partial saturation conditions. This shows that roots elongate more in partially saturated systems in search of water. Finally, in Fig. 2f, we measure primary root length, *L*_*P*_, with respect to the vertical distance between primary root tip and the surface, *L*_*v*_. The solid line represents *y* = *x*, which corresponds to the case when the root grows in a straight vertical manner perpendicular to the surface. Due to the presence of granular particles, root growth is never straight so any deviation from this solid line can be seen as a measure of the root’s tortuosity. Roots in partially saturated systems grow deeper and deviate less from the line, i.e. they grow in a more straight manner than the roots in the fully saturated system. This observation suggests the necessity for roots to find areas of higher saturation using the shortest possible distance.

### Modifications in water distribution due to structural changes in granular medium

From experiments, it is evident that the roots are never able to grow sufficienty fast enough to reach the fully wet region at the bottom of the cell. It is therefore imperative to redistribute water from the more saturated area at the bottom of the cell back to the root zone. During evaporation, hydraulic networks in the PSZ formed from liquid film connections or coalescence of liquid bridges already drive water upward via capillarity^12,31–35,41^ but these networks eventually disconnect as liquid films gradually thin out as evaporation of water continues. Thus, to replenish these films, we must constantly induce capillarity even as the water continues to evaporate. Classical root studies have noted little evidence that capillary rise can be effective at supplying water to the roots^43^. However, at local levels at the vicinity of the root, capillarity can play an important role in water absorption. This occurs at spatial scales up to millimeters and some studies have even reported water uptake up to centimeter spatial scales from the root tip^10,11,44^, which have also been confirmed experimentally via neutron radiography^45^. Water redistribution via capillary action from the more saturated bottom layer to the less saturated top layer is similar to hydraulic lift phenomenon^36–39^ - the movement of water from moist to dry areas^27^ through the roots. In our case, we redistribute water using an inhomogeneity in the granular medium.

**Figure 3:**
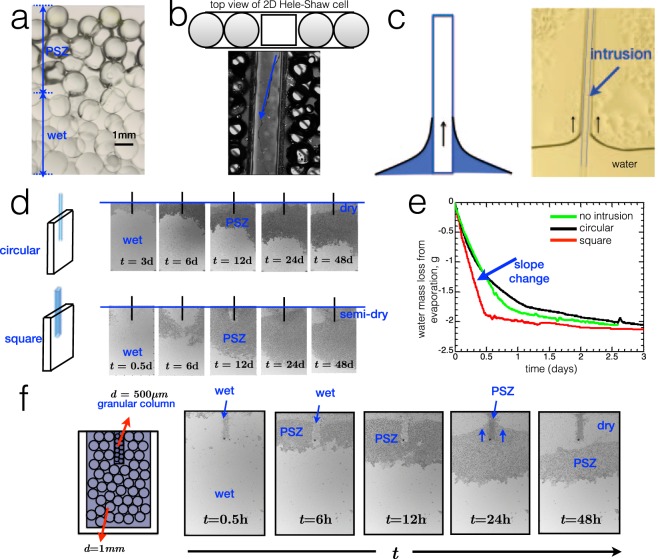
(**a**) Magnified image of granular medium made of glass beads with PSZ and fully wet region. There is a difference in meniscus volume along the vertical direction that induces capillary pressure. (**b**) Experimental image showing water flow along the length of the inserted intrusion. (**c**) Illustration of the continued capillary rise in the vicinity of the intrusion when inserted in the 2D cell filled only with water (no beads). (**d**) Experimental series of images showing the evolution of the front and the water distribution in the presence of two intrusion geometries, circular and square. (**e**) Water mass loss curve (evaporation) for the 2D porous medium with insertions of two different geometries of the solid rods. These are compared to the mass curve obtained for a typical 2D cell without intrusion. In these experiments, the size of the beads is *d* = 500 *μ*m. (**f**) Illustration of the 2D wet granular heterogeneous media consisting of a granular column of *d* = 500 *μ*m surrounded by *d* = 1 mm. Experimental images of evaporation experiments of the same system showing a saturated column of *d* = 500 *μ*m while a PSZ develops in the medium of *d* = 1 mm.

The image in Fig. 3a shows a portion of granular medium with the PSZ and fully wet region. The volume difference between the upper and lower parts in the porous medium helps drive capillary action. The insertion of a solid intrusion that spans the vertical length of the cell can help maintain a liquid film upwards. This intrusion is in the form of a square rod whose thickness is slightly less than of the gap of the 2D growth cell, generating a tiny narrow gap of characteristic small size as shown in Fig. 3b between the intrusion and the wall of the growth cell permitting capillary action of a thin liquid film. Even without the presence of a granular material, thin rods surrounded with water induce liquid to rise in the vicinity of the rod due to capillary action^46^, shown schematically and experimentally in Fig. 3c. The addition of the intrusion modifies the structure of the soil and induces a gradient of water distribution through wetting. As water evaporates, capillary action along the intrusion drives water from the deeper and more saturated parts of the soil to the upper part of the soil to replenish the quantity of water lost from evaporation. As the PSZ continues to develop due to evaporation further inside the medium, water is also pumped near the intrusion.

Images in Fig. 3d show an evaporation experiment in the presence of an intrusion. The square rod modifies the water distribution, which results in an initially non-horizontal formation of the PSZ, keeping the area near the intrusion saturated. This is not the case for the standard circular intrusion. Mass curves in Fig. 3e show that total water mass loss remains roughly the same with or without intrusion but the form of the curve does change notably in the slope during early evaporation times. The steeper slope suggests that the square rod introduces another significant pathway for water transport. This effect is not observable with a typical circular capillary intrusion, where the PSZ develops in a typical manner as if the intrusion was absent. Mass loss curves for the circular capillary also do not show significant difference with respect to mass loss in a standard 2D cell without intrusions. Results suggest the the presence of the square intrusion modifies water redistribution in the medium. These experiments were performed using different monodisperse homogeneous glass beads of *d* = 500 *μ*m and *d* = 1 mm at different insertion depths.

We also perform another experiment, in Fig. 3f, by structurally introducing another inhomogeneity in the form of different bead sizes. But instead of mixing them to yield a polydisperse system, we artificially create a column of *d* = 500 *μ*m beads surrounded by *d* = 1 mm beads. The smaller pores of the 500 *μ*m-bead column with respect to the surrounding larger pores will generate a pressure difference that results to capillary pumping. This effect, in some ways, reflects that of the capillary action induced by the solid intrusion. As the PSZ develops from evaporation, a pressure gradient exists along the capillary liquid films between the bottom and top part of the PSZ, which drives upward flow of water^12,31–35,41^. The PSZ contains air-liquid interfaces whose curvature drives a pressure distribution along two points in the liquid network. In the initial image, the 500 *μ*m-bead column appears dark because it is not a monolayer as the thickness of the cell is essentially set by the diameter of the larger beads. As time progresses, it changes hue due to the contrast of the refractive indices between the surrounding PSZ. Although the actual saturation content in the 500 *μ*m-bead column was not measured, it is a reasonable assumption to make that at early times, the column remains roughly fully saturated as water from the larger pores evaporate first. The capillary effect is eventually seen at longer times (e.g. *t* = 24h) as the 500 *μ*m-bead column perturbs the front and liquid rises near the column. The intrusion in the form of a granular column has higher water saturation relative to the rest of the medium because the smaller pore sizes have higher capillary pressure allowing them to more strongly hold water. Evaporation studies involving coupled heterogeneous systems^47^ have shown that water from the larger pores is de-saturated first prior to the smaller pores.

### Preferential tropism and changes in root morphology due to structural inhomogeneities

**Figure 4:**
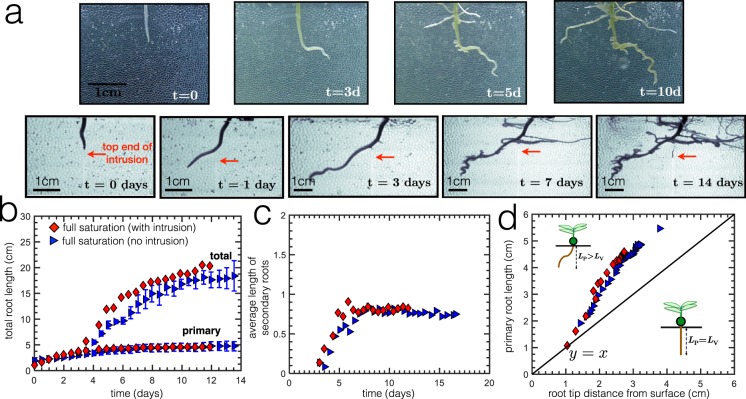
(**a**) Experimental images of root growth under full saturation conditions with and without intrusion. (**b**) Comparison of primary and total root length with and without intrusion under full saturation. (**c**) Average number of secondary roots normalized by the number of branching points. The inset figure shows an experimental image at *t* = 14 days, where the root still does not find the intrusion under full saturation. The scale bar is 1 cm. (**d**) Root length as a function of tip distance. The solid line is *y* = *x* suggests that the root grows in a straight vertical manner perpendicular to the surface. Results show that under full saturation, the intrusion does not significantly change the root elongation and root morphology.

We first perform experiments with and without intrusion under full saturation conditions, shown in Fig. 4a. For these experiments, we perform at least three runs. When water is constantly replenished throughout the experiment, results show that roots do not find the intrusion. The graph in Fig. 4b demonstrates that root growth (both primary and total) in the presence of the intrusion is comparable to that without the intrusion. Thus, the intrusion under full saturation does not induce a significant effect and root morphologies are similar, even the average length of the secondary roots as shown in Fig. 4c. In Fig. 4d, the deviation of the primary root from the vertical line *y* = *x* is also similar for both experiments, thereby also suggesting similar root morphologies under full saturation conditions. The constant presence of water might also hinder growth due to hypoxia and insufficient quantity of oxygen^48^. This might explain why quite a number of secondary roots are clumped near the top part of the root and pointing upwards, possibly in the direction of the surface in search of oxygen.

**Figure 5:**
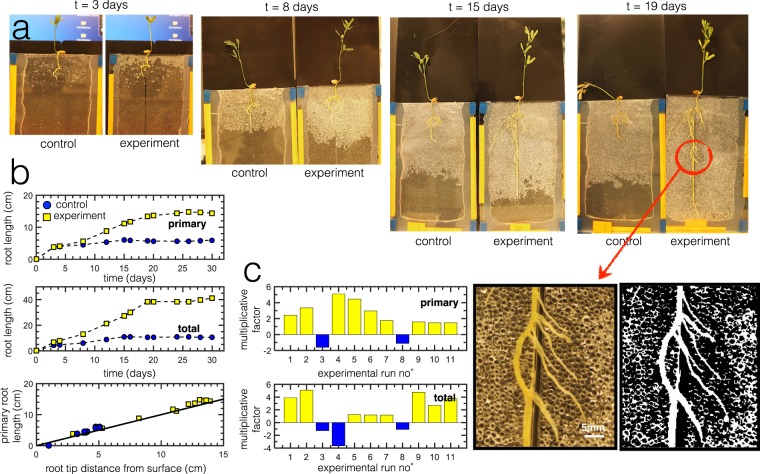
(**a**) Typical example of time evolution of root growth experiment with intrusion (experimental) and without intrusion (control). Both experiment and control set-ups are performed simultaneously under partial saturation conditions. Results show a change in root morphology and a distinct vertical growth pattern in the presence of the intrusion. A magnified image of a portion of the root shows the deviation from the intrusion before briefly returning towards it. Treatment of water content (white) around the area shows minimal water near the secondary roots that have sprouted across the intrusion. (**b**) (top) Primary root growth curve for the images in (a). (middle) Total root growth curve (primary + secondary) for the images in (**a**). (bottom) Primary root length as function of root tip distance from suface. The solid line represents *y* = *x*, which corresponds to the case when the root grows in a straight vertical manner, perpendicular to the surface. In the presence of the intrusion, the root follows a vertical growth pattern. (**c**) (top) Bar graph showing the primary root length for experiments with intrusion compared to those without intrusion. A positive multiplicative factor shows that for a particular experimental run, the root length with intrusion is longer than without intrusion by the value of the factor (e.g. the primary root length in Run no° 1 is nearly twice longer than without intrusion). Conversely, a negative factor simply indicates the opposite, i.e. the root length without intrusion is longer than with intrusion (e.g. Run no° 3). (bottom) Bar graph showing the total root length for experiments with intrusion compared to those without intrusion.

Then, we perform experiments with and without intrusion under partial saturation conditions. For these experiments, we perform a total of eleven experimental runs. In comparing control and experimental set-ups, the final root branching architecture is also another aspect to consider^10,49^. Experimental results in Fig. 5a show the root morphology in the presence of the solid intrusion. The evidence comes from the vertical growth pattern of the primary root that is typically absent without the intrusion. The vertical growth pattern occurs adjacent to the intrusion and the root exhibits preferential tropism towards the intrusion. As the PSZ recedes due to evaporation, the effect of the intrusion also guides the roots deeper and allows them to stay within a PSZ, permitting the roots to proliferate further. Here, we define “preferential tropism” as a general type of root response that favors a certain growth direction due to what it perceives as changes in its surroundings. In this case, one may argue that the local granular packing adjacent to the intrusion has ~30% more void space and the resulting decrease in mechanical impedance takes precedence. However, results consistently show that this preferential tropism is a combination of hydrotropism triggered by the robust capillary action in the vicinity of the intrusion and gravitropism once the root reaches the intrusion as the intrusion guides the roots downwards. The magnified image shows the primary root initially deviating from the intrusion probably unable to push through the packing but then returns to the intrusion. Secondary roots then grow towards the intrusion as evidenced by the minimal water content around the root area in the treated image. Due to the narrow gap between the intrusion and the 2D cell wall, the roots seem to squeeze through this tiny area. This depicts the ability of the roots to physically adapt to their physical environment.

General analysis of root elongation, typically such as in Fig. 5b (top), shows that the primary root grows much longer with intrusion, guiding the roots deeper and towards more saturated areas of the medium - something that is difficult for the roots to achieve without the intrusion. In Fig. 5b (middle), the total root growth also is in favor of the presence of the intrusion. The graph in Fig. 5b (bottom) shows that the growth of the primary root is nearly vertical and straight, almost parallel to the inhomogeneity. Because the roots with the intrusion have greater access to water in response to the stress induced by evaporation, they generally thrive longer based on plant lifetimes. Plant lifetime was determined from experimental images, where a qualitative deterioration of plant shape and form (dehydration, wilting, etc) suggests it is already dead. We measure final root length before death. In Fig. 5c (top), we compare the primary root length and in Fig. 5c (bottom), we compare the total root length. This graph is divided into two sections expressed in multiplicative factors, i.e. a positive factor shows that the root length (primary or total) with intrusion is greater than without intrusion given by the value of the factor. In contrast, a negative factor simply shows the opposite, i.e. the root length without intrusion is recorded to be greater than with intrusion for that particular experiment. In most cases, results show that growth with intrusion is always greater than without intrusion. In one case (Run no° 4), the experimental cell with the intrusion records a longer primary root but shorter secondary roots while the control cell has a shorter primary root but longer secondary roots. This explains why the total root length is longer without the intrusion. The development of secondary roots is linked to the need of the roots to search for more saturated areas^50^.

In general, the presence of the intrusion increases root length further into deeper sections of the 2D soil and, by extension, also increases overall plant lifetime. When evaporation of water is allowed to naturally take its course under partial saturation conditions, the presence of a PSZ seems favorable for the root. This could be attributed to the presence of the oxygen in the air-water mixture of the PSZ. Equally, the decreasing water content could also induce stress in the roots, thereby permitting them to adapt by growing towards areas of relatively higher saturation. Thus, the observed phenomenon is not only due to the roots following the barrier and that the intrusion has two roles. First, it creates a humidity gradient that elicits a response from the root to grow towards it. Then, the intrusion further guides the roots towards the deeper parts of the soil. While roots generally prefer to cluster in macropores^22^, we have nevertheless shown that roots can squeeze through tiny spaces in search of water.

### Root behavior induced by inhomogeneities

**Figure 6:**
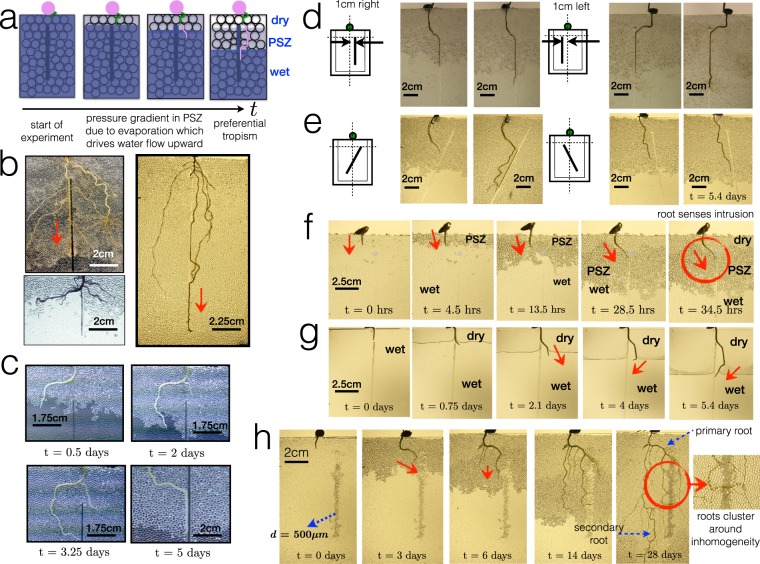
(**a**) Schematic of the root deviation and preferential tropism with respect to changes in water distribution in the granular medium. As the water saturation decreases due to evaporation, the roots experience stress and grow towards the intrusion. (**b**) Experimental images of root growth showing preferential tropism adjacent to the intrusion. In most cases, it is the primary root that grows towards the intrusion but secondary roots have also been observed to do the same. (**c**) Experimental image an initial deviation of root elongation away from the intrusion but later finds it when the PSZ continues to develop. (**d**) Experimental images of root growth showing preferential tropism of intrusion at varying distances and orientations. Intrusions are oriented vertically but 1 cm both left and right from the center. (**e**) Intrusions are oriented diagonally. For these experiments, images are taken on average every 3 days. (**f**) Time evolution images focusing on the first few hours of root growth with evaporation. As the PSZ develops, the root exhibits a sharp bend towards the intrusion. (**g**) Time evolution images of root growth in an empty cell with intrusion. Roots exhibit a sharp bend and eventually grow towards the intrusion as soon as the evaporating front catches up with it. Arrows indicate direction of growth. (**h**) Experimental images of root growth with another type of inhomogeneity - a granular column made of smaller pores. Some roots initially cluster around the granular column even penetrating through the tiny pore spaces.

The scheme in Fig. 6a depicts preferential tropism with respect to changes in water distribution. Additional images in Fig. 6b show the reproducibility of the effect of the physical intrusion on total root growth. In some experiments, such as in Fig. 6c, roots initially deviate away from the intrusion probably due to physical limitations (e.g. unable to sufficiently push granular particles aside). However, the roots later find the intrusion. Repeated experimental results show that the roots consistently grow towards the intrusion while the rest of the medium around it is being de-saturated with water from evaporation. As a result, the roots elongate in the direction of the intrusion and grow adjacent to it.

Thus far, the seed has relatively easy access to the intrusions due to the intrusion being positioned not far beneath it. Sometimes, as seen in Fig. 6c, the roots can initially deviate before finding the intrusion. This raises the question of characteristic distance: how far must the seed be from the intrusion to still be able to sense the intrusion? To offer insights into the question, we perform experiments by deliberately varying the distance of the intrusion about 1 cm away on either side from the initial root as shown in Fig. 6d. Results consistently indicate that the root continues to develop in the direction of the intrusion, assisted principally by the growth of its secondary roots. We have also performed a modified version of the problem by putting the intrusion in an oblique position shown in Fig. 6e. In this manner, we demonstrate competition between capillarity along the intrusion wall and gravity. Results once again show consistency of preferential tropism towards the intrusion despite the less favorable position of the intrusion. This suggests that while roots generally respond to gravity, they prefer to grow towards areas of higher saturation^22^.

In one experiment, Fig. 6f, we focus on the early hours of root growth and evaporation. Here, the root is initially positioned 1 cm away from the intrusion but the root exhibits a sharp bend towards the intrusion especially when water evaporates.

In another experiment, we remove the granular material and simply let the roots grow in an empty medium with the intrusion. Results in Fig. 6g show that as the root is still submerged during the early times, the root does not grow towards the intrusion but exhibits a sharp bend at *t* = 4 days towards the intrusion when the front begins to catch up with the root.

Finally, we perform an experiment coupling a 500 *μ*m-bead granular column with root systems. Images in Fig. 6h, suggest that the roots initially grow in the direction of the column as the PSZ develops during evaporation. Secondary roots start growing only once the primary root has reached the column. At later times, root systems cluster around the granular column suggesting active root response to higher water content in that area contained in the smaller pore spaces, where roots could have possibly used the water from the granular column to fuel the emergence of lateral roots to other areas. The results reinforce the idea that roots definitely respond to the presence of inhomogeneities (granular column) and to areas of higher water content. The granular column “stores” water in its small pore spaces in the same vein as the solid square intrusion induces liquid flow via capillarity. From images, the primary root still develops adjacent to the column showing preferential tropism, while launching smaller secondary roots that cluster around the intrusion. Plant roots possess a certain degree of adaptability to environmental changes by initially using their foraging organs (roots) in search of areas of high concentration of resources^50^. Furthermore, the inset image shows that roots are capable of growing into tiny pore spaces and does not necessarily always prefer to grow in larger pores.

## Conclusion

Inhomogeneities in granular media have shown to actively and physically influence root elongation caused by the redistribution of water content in the medium. The results are similar to observed hydraulic lift where water is passively redistributed by the roots from a moist to a dry area. In these experiments, however, this transport is induced by the structural intrusion. The effect cannot just be attributed solely to the looser packing adjacent to the intrusion but equally important is the robust liquid film being pumped along the intrusion while the rest of the medium loses water via evaporation. Thus, the role of the intrusion is two-fold. First, it pumps water from the bottom towards the top creating a gradient that elicits a response from the root to grow towards it. Then, the intrusion further guides the roots towards the deeper parts of the soil.

The capillary action along the intrusion is a primary result of the 2D nature of the set-up and depends on the small contact area between the intrusion and cell wall. From a fundamental perspective, it would be interesting to understand how a change in the size of this contact area affects preferential tropism and, in addition, how such 2D effect can be translated into real 3D systems. On the other hand, from an applications perspective, root elongation is important for increasing plant lifetime, which also improves agricultural yield. Future studies must focus on how root elongation adapts with respect to different levels of water saturation in the medium. This serves as a jump-off point for designing better additives that not only store water but also guide roots to more saturated regions inside the medium; thereby maximizing water usage and improving efforts to increases plant lifetime - key aspects in applications involving water retention.

## Materials and Methods

### 2D growth chambers

The root growth cell is built from two glass sheets of size 10 cm (length) and 15 cm (width). All the sides, except the upper part, are sealed with commercial silicon paste. Due to the presence of the paste, the effective dimension of the porous medium is reduced to about 7.5 cm × 13 cm. To ensure that the cells contain a monolayer stack of glass beads, *d* = 1 ± 0.2 mm (borosilicate, Sigma-Aldrich, USA), we a put a spacer with a thickness approximately equivalent to the diameter of the glass beads. The glass beads initially have around 20% polydispersity, but are sieved prior to the experiment to only use monodisperse sizes as possible. Despite the sieving process, the cell is still quasi-2D since a perfect 2D system cannot be achieved. We then wash the glass sheets with hydrophobic silane solution (OMS Chemicals, Canada) for two hours to reduce wetting effects along the glass wall. The glass beads are all hydrophilic for all root growth experiments, washed with 0.1 M HCl and dried in an oven overnight at 70 °C. Contact angle values are 82 ± 4° for hydrophobic glass sheet and 16 ± 4° for hydrophilic glass beads.

### Root growth set-up

Root growth studies are commonly performed through monitoring a series of changes in root elongation as a function of time. However, they can be challenging because roots naturally grow in opaque or subsurface systems^5^. While 3D imaging systems such as X-ray tomography or neutron tomography have been gaining traction in the community^45,51,52^, there are generally hardly accessible and are almost always expensive. Thus, root investigations have often relied on simple set-ups, normally in two-dimensions (2D). These techniques called rhizotrons^11,21^ permit non-invasive spatial assessement of the distribution of the root as it develops in the granular medium. The method, when back-illuminated with a light box, also potentially permits simultaneous observation of water content over time. For this reason, the choice of granular material is often transparent (e.g. glass beads).

We characterize root growth in such rhizotron systems, where we let the roots grow under a controlled environment (*T* = 23° ± 2, *R*_*H*_ = 45.0 ± 5.0%). We use lentils (*L. culinaris*), which have also been previously grown in 2D growth cells^21^ and results show that they still exhibit robust growth and that their growth in 2D model systems are still comparable to that of real soil systems in 3D^20^. The lentil seeds are first allowed to germinate in moist paper or tissue in the dark for 2–3 days. Once a radicle has developed from the seed, it is then ready to be transplanted on top of the 2D growth cell. The initial condition of the cell is full water saturation. This means that Φ = 1 at *t* = 0, where *t* is time. To fully saturate the growth cell, we use a vacuum water pump by entirely submerging the cell in a water bath filled with the liquid. This vacuum pump apparatus simply removes air from the medium, allowing the surrounding water to percolate and enter the medium. Once the cell is completely saturated, we then place the germinated seed on top of the 2D cell. The average length of the radicle that has sprouted at the time of seed transplant onto the top surface of the Hele-Shaw cell is *L*_*T*_ = 1.5 ± 0.5 cm. It is expected that the initial phase of root growth would be normally slow^40^ since roots are still developing the necessary biological functions dedicated to certain root processes. This is the initial lag phase^40^ and during this time, water loss is being regularly replenished. After the lag phase, we perform experiments either under conditions of full saturation, where water loss from evaporation is constantly being replenished, or under conditions of partial saturation, where evaporation is allowed to take its course. The water used actually contains a small amount of Hoagland solution (1/4 volume ratio) to provide the necessary nutrients for plant growth. Nutrients are especially crucial during the developing phase of the seeds. The plants were constantly grown under grow lights with light intensity of ∼40 *μ* mol/m^2^s. Some types of lentils use a light intensity of ∼110–150 *μ*mol/m^2^s, but for *L. culinaris*, similar studies have employed comparable light intensities (e.g. 37 *μ*mol/m^2^s^21^). It might be considered low light but is actually suitable for our experiments.

### Image acquisition

We take images of root growth as function of time generally using Canon 500D SLR camera at certain intervals since root elongation time scales are normally slow. We use 18–55 mm lens and the spatial resolution was about 35 *μ*m per pixel. We use a light box to back-illuminate the 2D cell to completely distinguish the main components: roots, beads, and water. Water content can be estimated from intensity of a transmitted light^10^. However, the resolution does not permit accurate assessment of water saturation using this technique.

The obvious challenge when studying root systems is that the duration of the experiment usually lasts relatively long. For lentils, growth characterized by a fully developed root and shoot system is observed within 12–18 days. In addition, experimental investigations on root systems need to be performed several times to ensure that an observed phenomenon is indeed robust and reproducible. The slow time scales involved with studying root growth normally requires simultaneous experiments to obtain as much results as possible within a limited time frame. The lack of multiple Canon 500D SLRs required use of commercial webcams as supplementary imaging tools. These webcams have been automated using a Matlab program.

We measure root length via the segmentation method. The methods breaks down a continuous curve into smaller line segments, whose individual lengths have been calculated from a pixel-to-length calibration that is performed before each measurement. Root length measurement via image substraction is often difficult to perform with these experiments because sometimes the thin secondary lateral roots in the PSZ are too faint to be detected. Roots in granular systems normally adopt a curved profile. As a result, the total contour length is measured by creating smaller line segments. Preliminary tests of the segmentation method performed on various sets of curves of known lengths reveal a measurement error of ±5%. The total length is measured from the summation of the lengths of the individual line segments.

## Data Availability

The datasets generated during and/or analyzed during this study are available from the corresponding author on reasonable request.
